# Supplementary material for: Förster resonance energy transfer and protein-induced fluorescence enhancement as synergetic multi-scale molecular rulers
Source: Sci Rep. 2016 Sep 19;6:33257. doi: 10.1038/srep33257 (PMC5027553; doi:10.1038/srep33257)
Supplement: Supplementary Information [file srep33257-s1.pdf]

Supporting Information for the manuscript

Fluorescence resonance energy transfer and protein-induced fluorescence enhancement as synergetic multi-scale molecular rulers

Evelyn Ploetz[1,#, §], Eitan Lerner[2,#], Florence Husada[1], Martin Roelfs[1], Sangyoon Chung[2], Johannes Hohlbein[3,4], Shimon Weiss[2] and Thorben Cordes[1,]*

[1] Molecular Microscopy Research Group, Zernike Institute for Advanced Materials, University of Groningen, Nijenborgh 4, 9747 AG Groningen, The Netherlands

[2] Department of Chemistry and Biochemistry, University of California Los Angeles, Los Angeles, CA 90095, USA

[3] Laboratory of Biophysics, Wageningen University and Research Centre, Wageningen, The Netherlands

[4] Microspectroscopy Centre, Wageningen University and Research Centre, Wageningen, The Netherlands

[§] Present address: Physical Chemistry, Department of Chemistry, Ludwig Maximilians-Universität München, 81377 Munich, Germany

# these authors contributed equally; *corresponding author, t.m.cordes@rug.nl

ADDITIONAL DATA AND FIGURES

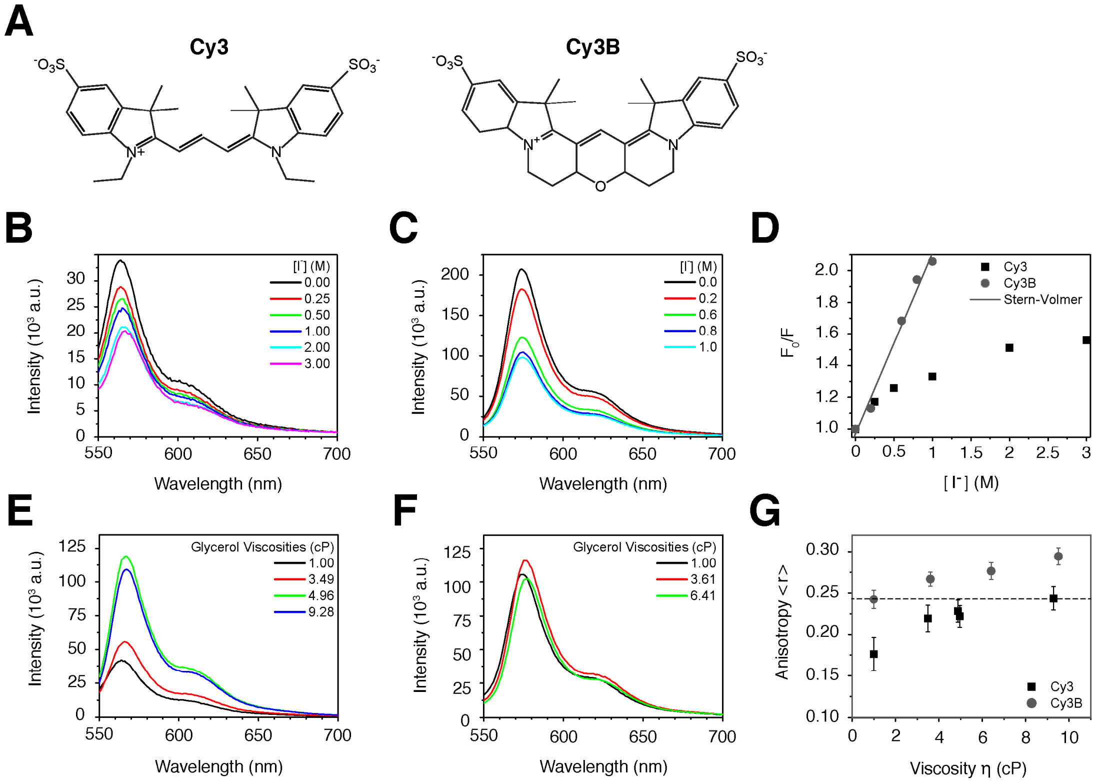

Supplementary Figure 1. Photochemical properties and characterization of the Cyanine dye Cy3. (A) Chemical structures of Cy3 and Cy3B. **(B-D) Chromatic changes due to quenching.** Bulk fluorescence measurements of free lacCONS+20A promoter dsDNA with Cy3(B) in the presence of Iodide. Panels (B) and (C) demonstrate the change in Cy3(B) fluorescence due to Iodide collisional fluorescence quenching to be at the same Iodide concentration scale. (F) Stern-Volmer-plots of Cy3(B) collisional fluorescence quenching. While Cy3B has a typical linear Stern-Volmer-plot, Cy3 has a nonlinear one characteristic of a system having fluorophores accessible and inaccessible to the iodide quencher[1]. **(E-F) Influence of microviscosity on cis-trans isomerization seen in bulk experiments.** Steady state fluorescence measurements of free lacCONS+20A promoter dsDNA with Cy3(B) in the presence of glycerol. Panels F and G show the change in Cy3(B) fluorescence due to viscosity to be mostly intensity changes but little chromatic changes. **(G)** Steady state fluorescence anisotropy values for increasing buffer viscosity. The QY increase in Cy3 is directly related to rotational freedom, which is not found for Cy3B.

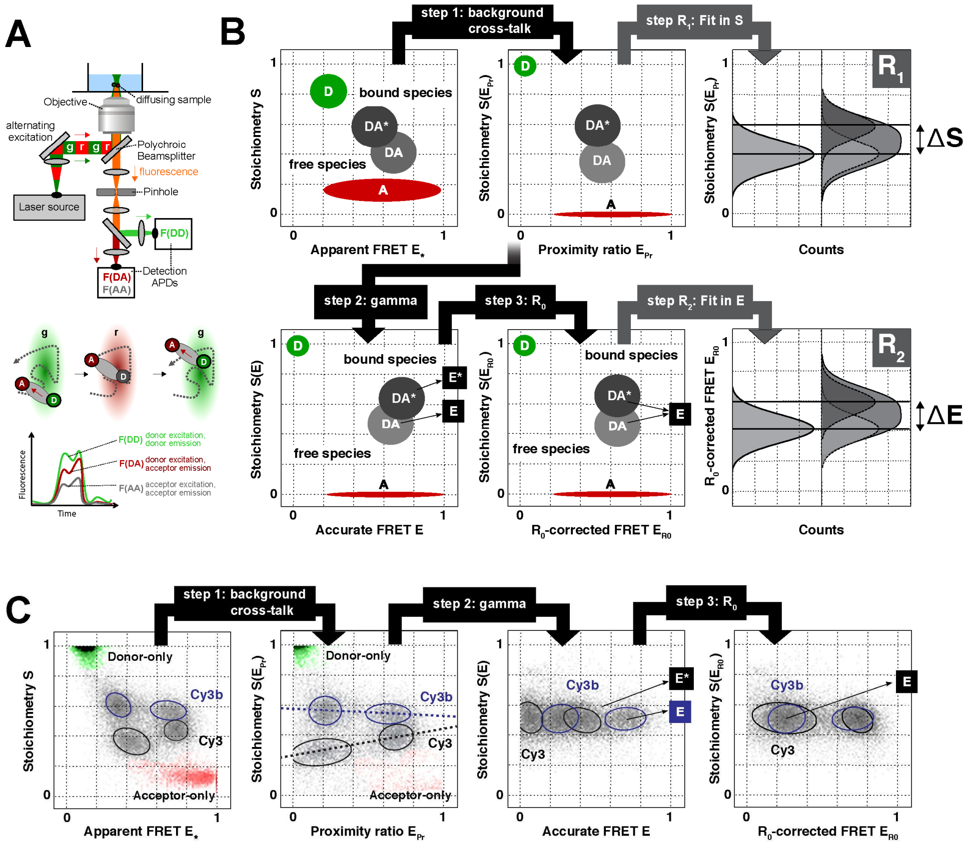

Supplementary Figure 2. Data analysis in PIFE-ALEX. (A) Alex-Spectroscopy. Schematic of a confocal ALEX-setup working in solution with alternating laser-excitation. During the transit of labelled biomolecules through the focus, the periods of green (g) and red (r) excitation alternate faster than the corresponding diffusion time. Upon direct excitation of the donor, energy can be transferred to the acceptor via FRET. The existence of the acceptor is probed during red excitation. As a result, every burst is characterized by three photon streams (F(DD), F(DA), and F(AA) that allow to calculate FRET E* and stoichiometry S per burst. Both values allow to construct two-dimensional histograms and separate different species according to S (see panel **B**): donor-only (S=1, green), acceptor only (S=0, red) and double labelled molecules (grey).**(B) Work-flow.** Labeled molecules are sorted in a 2D histogram according to apparent FRET efficiency E* and labeling stoichiometry S: Donor-only specie (D) around S=1, Acceptor-only specie (A) around S=0 and double-labeled molecules in-between. Double-labeled, free sample (DA) and protein-bound sample (DA*) are discernible by a shift in S. **Short distance R_1:** To determine the short distance R_1, the 2D-data is corrected against background and crosstalk (step 1), populations are fitted with 2D Gaussians and projected on the corrected stoichiometry axis (step R_1). The difference in S reports on R_1. **Long distance R_2:** After background and cross-talk-correction (step 1), each population is corrected with its own gamma-value (step 2) (For details see methods section/Supplement). Since the fluorophore-pair experiences a different environment in the free and complexed form, the altered Förster radii need to be converted onto a common reference (step 3). Having one Förster radius in common, the populations are fitted with 2D Gaussians and projected on the R_0-corrected FRET axis (step R_2). Their FRET values/difference in E can now be linked to true distances/distance changes in R_2. **(C)** Demonstration of data analysis for Cy3(B)-ATTO647N labeled dsDNA with 13 and 23 bp separation seen as 4 different population in the 2D-histogram before data correction. After background/crosstalk and gamma-correction for Cy3(B), all 4 populations line up at S(E) = 0.5. Due to differences in the Förster radius R_0 for the two different FRET-pairs, the accurate FRET values E are not identical yet. After R_0-correction, the accurate Fret values E_{R0} for Cy3(B)-ATTO647N coincide.

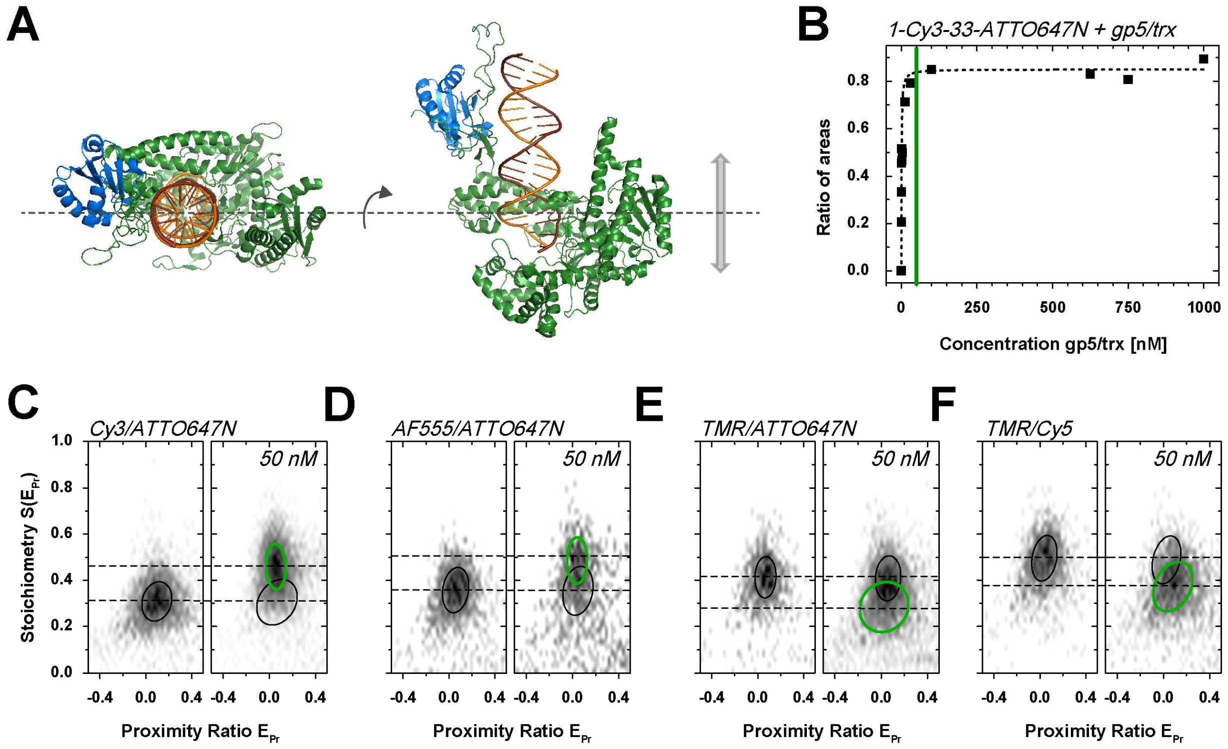

Supplementary Figure 3. Fluorophore specific PIFE effect due to non-specific binding of T7 DNA polymerase gp5/trx to dsDNA. (A) Crystal structures of *E.coli* T7 DNA polymerase *gp5/trx* (PDB:1T8E) **(B)** Titration curve monitoring the binding of gp5/trx to a 45bp-dsDNA labelled with Cy3 at the 5'-end and ATTO647n in 33bp distance. The apparent k_D is 0.7 nM. **(C-F)** PIFE-effect of gp5/trx on different FRET pairs attached to a 45-mer. While the donor is attached to the 5'-end, the acceptor is positioned in 33bp distance. **(C)** The enhancement of Cy3 is seen as a positive shift in Stoichiometry $S(E_{pr})$ for Cy3/ATTO647N. **(D)** For AlexaFluor555/ATTO647N, an enhancement of AlexaFluor555 is evident. **(E)** TMR/ATTO647N are similarly affected by gp5/trx. ATTO647N shows slight brightness increase for addition of gp5/trx. **(F)** Enhancement of Cy5 leads to a decrease in S as seen for the FRET pair TMR/Cy5.

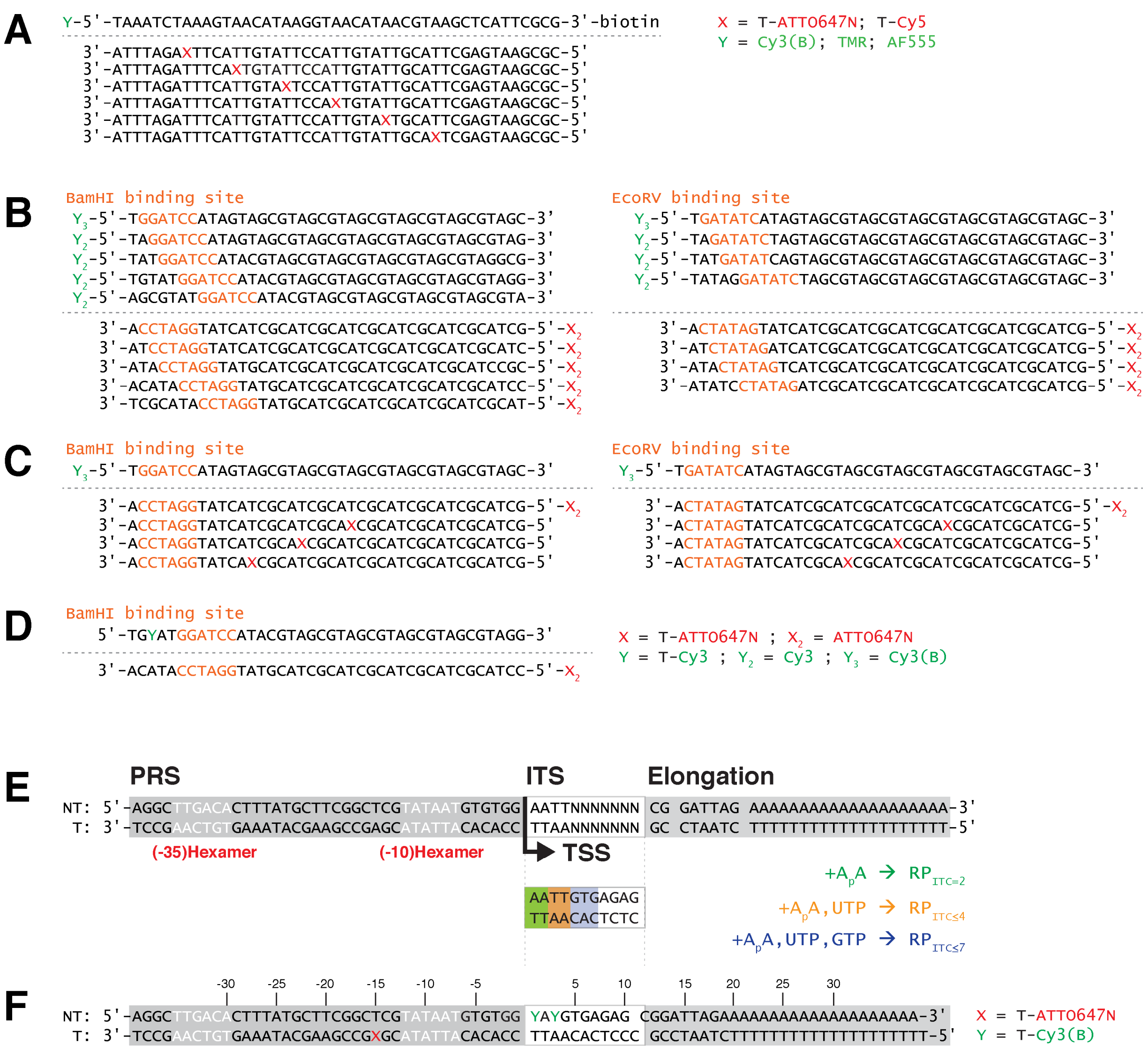

Supplementary Figure 4. Oligonucleotide sequences used in the present study. (A) DNA scaffolds labelled with Cy3(B) and ATTO647N to demonstrate the data correction procedure in Suppl, Fig. S2 and binding of gp5/trx. **(B-D)** DNA sequences including a palindromic sequence to the restriction enzymes *Bam*HI (left) and *Eco*RV (right). (E-F) lacCONS promoter sequences[2,3] including promoter recognition sequence (PRS), transcription start site (TSS), initial transcription sequence (ITS), the elongation sequence. Bottom part: ITS sequences that have been used to prepare the system in various RP_{ITC} states, and their corresponding NTP starvation mixtures. Donor and acceptor positions for the registers -15/+1 and -15/+3 are indicated.

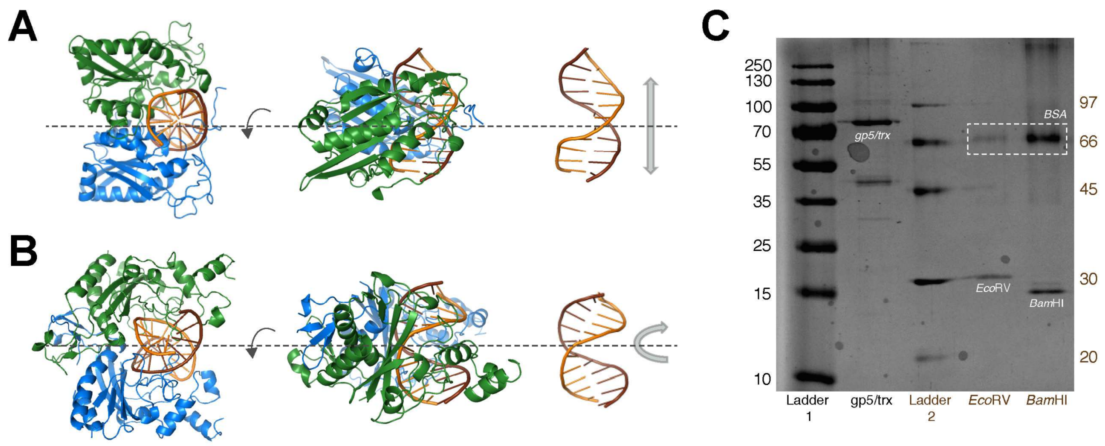

Supplementary Figure 5. (A) Crystal structures of the restriction enzymes *Bam*HI and *Eco*RV**.** Both DNA-binding proteins, *Bam*HI (PDB:2BAM) and *Eco*RV (PDB:1B94) have been crystalized in the presence of calcium and bind as dimer to dsDNA. They are among the bio-(physico)chemically best-characterized endonucleases. The presence of calcium inhibits their activity and prevents DNA cleavage via hydrolysis. (**A**) *Bam*HI forms a stable, pre-reactive complex on dsDNA containing the palindromic sequence GGATCC without changing the three-dimensional structure of the dsDNA. (**B**) Binding of *Eco*RV to the GATATC-site results in a tightly bound complex, which bends the DNA by 50°. **(C)** SDS-PAGE of purified DNA-binding proteins employed in this study. The T7 DNA polymerase gp5/trx complex has a molecular weight of 91.47 kDa, *Eco*RV has 28.66 kDa and *Bam*HI 24.57 kDa. No aggregates were observed.

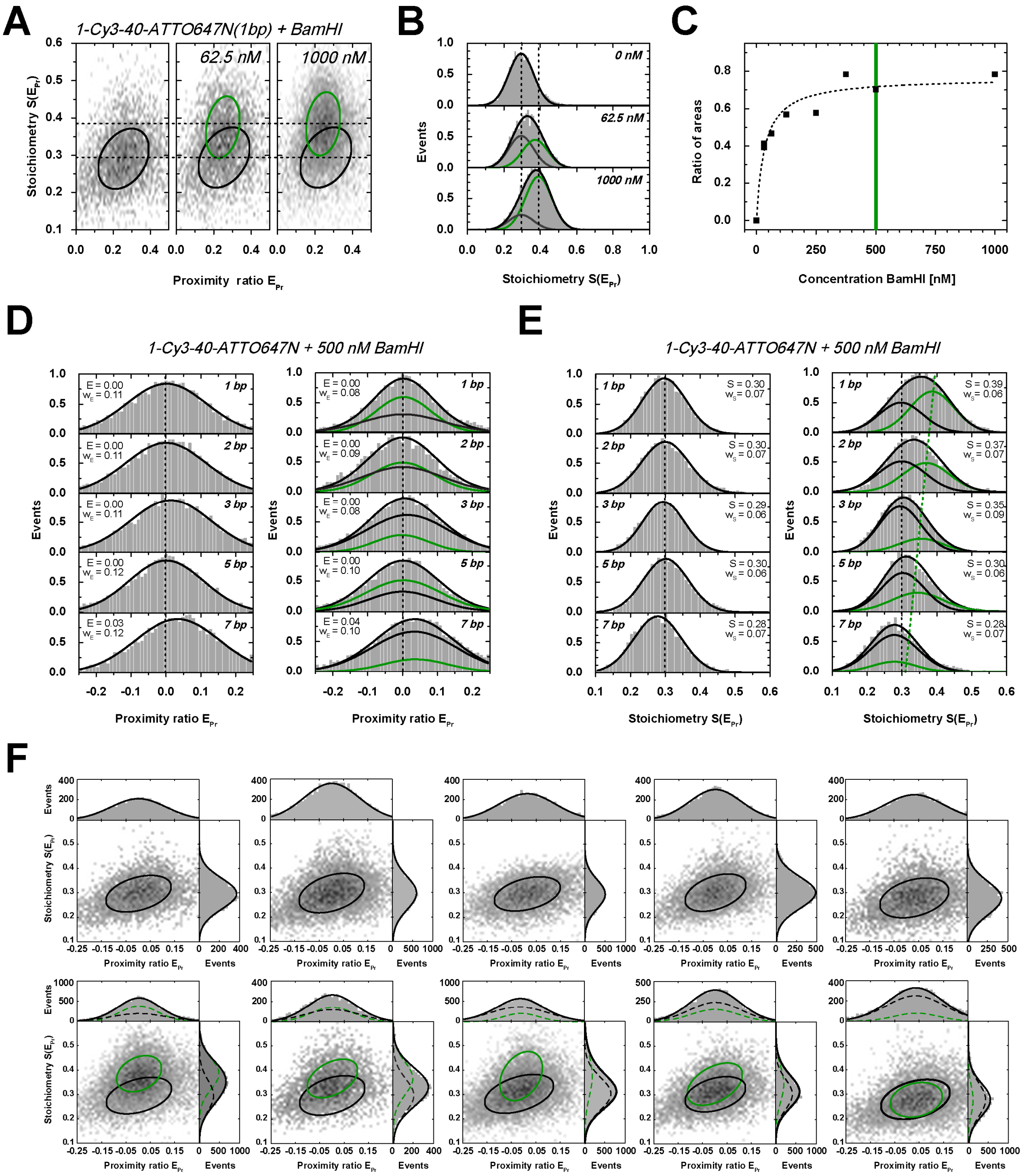

Supplementary Figure 6. Characterization of *Bam*HI as PIFE-ruler. PIFE between *Bam*HI and dsDNA labeled with Cy3 as donor fluorophore in 1bp separation. The acceptor ATTO647N is positioned on the opposite 5'-end in 40 bp distance. **(A)** Corrected 2D histograms of 40bp-dsDNA: Binding of *Bam*HI to free DNA results in a shift from S = 0.30 (unbound, black circle) to 0.39 (bound species, blue circle) for different concentrations. **(B)** Addition of *Bam*HI shifts the equilibrium of unbound to bound species as seen in a 1D-projection in S. **(C)** An apparent binding constant K_d of 37±8 nM was obtained from the ratio of volumes [bound/(unbound+bound)] between both populations, using two Gaussian distributions (see Online Methods). **(D-F)** Full data set of the *Bam*HI ruler presented in Figure 4D.

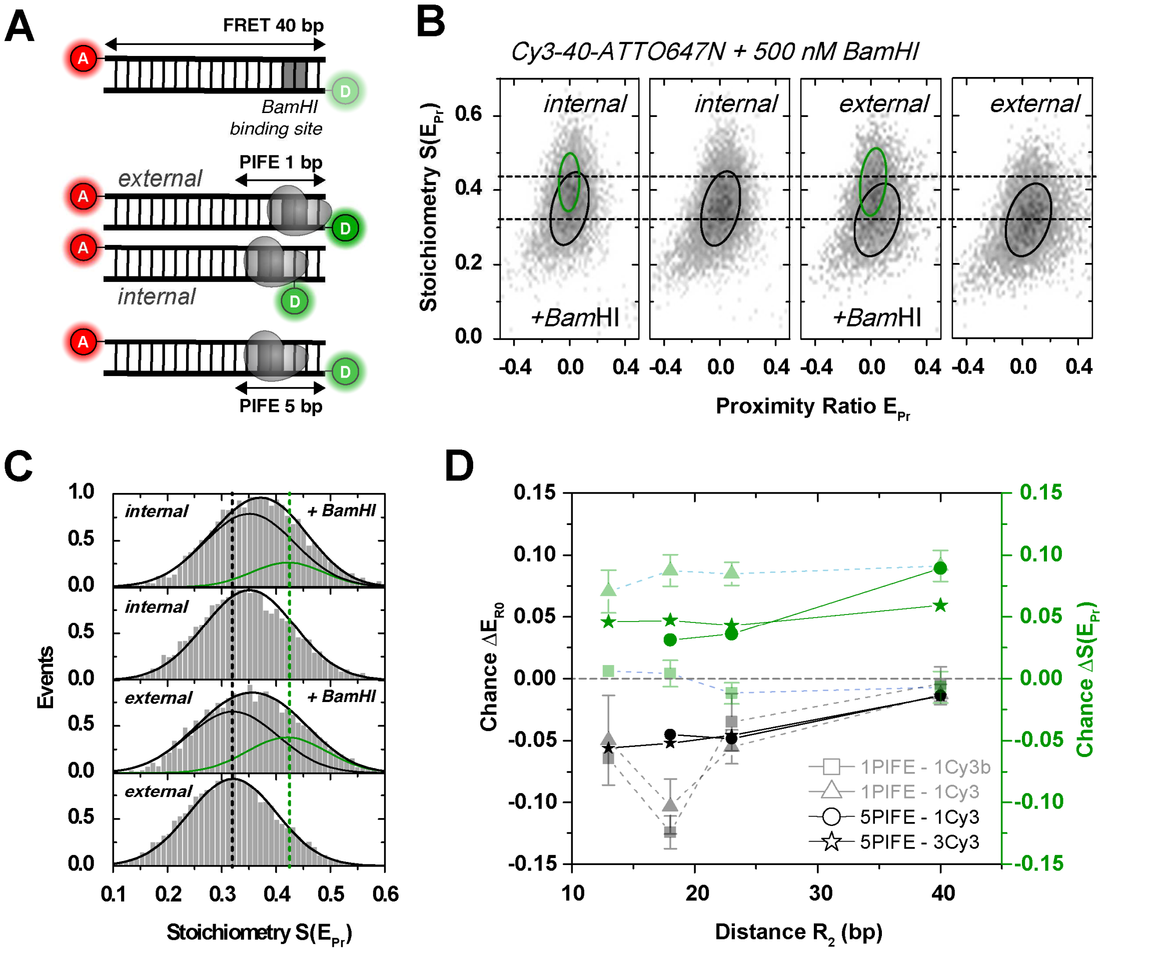

Supplementary Figure 7. PIFE effect of *Bam*HI for internal and external labelling of Cy3 on dsDNA. (A) At fixed acceptor position, Cy3 is positioned at the 5'-end (extern labelling) or at the 3rd base (internal labelling) pair of the opposite strand of a 40bp-dsDNA. The palindromic sequence is located in 1bp separation to the donor for both cases. **(B)** 2D histograms: free DNA (black circle) and DNA bound to 500 nM *Bam*HI (blue circle) for internal and external labelling. Base pair-stacking of Cy3 at the 5'-end leads to a decrease in fluorescence (S = 0.32) compared to internal labelling (S = 0.38). Binding of *Bam*HI is observed in an upward shift to S = 0.43 for both labelling schemes. **(C)** One dimensional projections of $S(E_{PR})$ data and 2D Gaussian fits and their centre positions (free form, black; protein-bound form, blue.

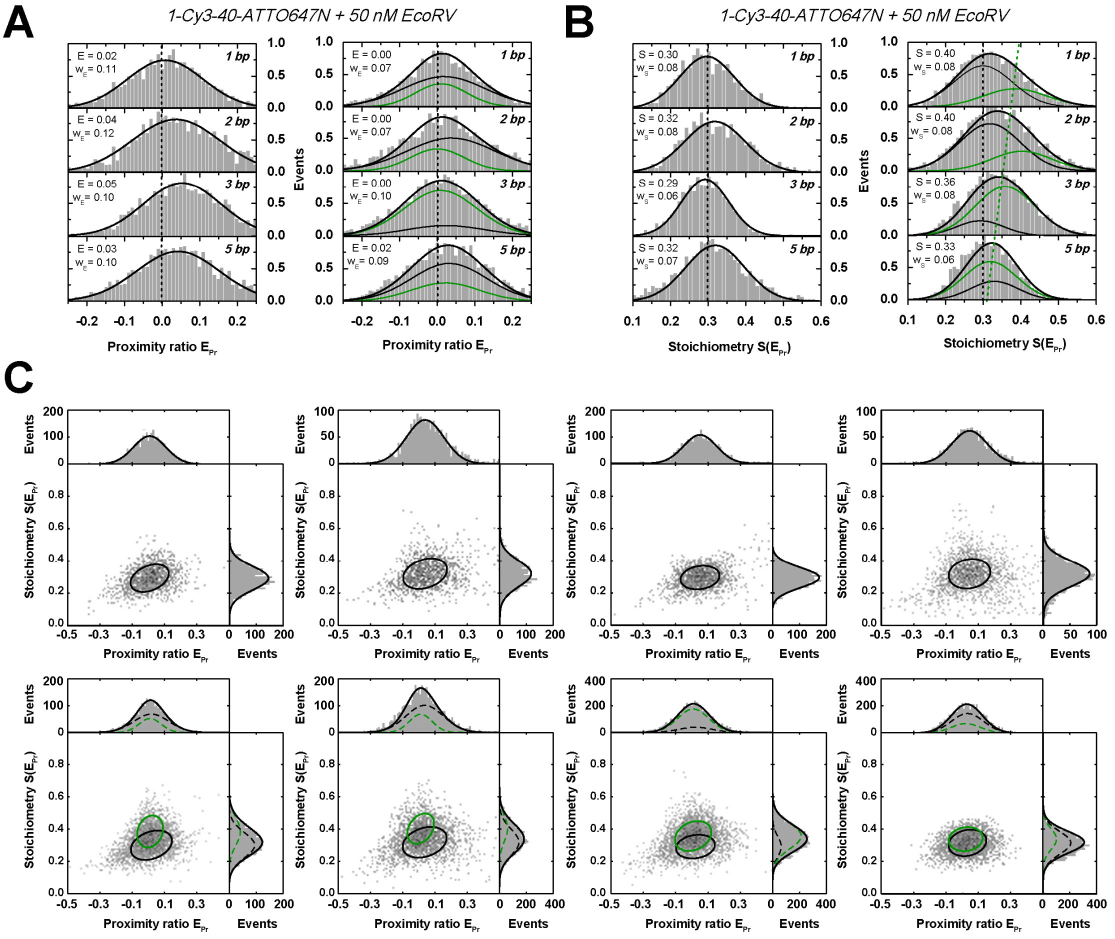

Supplementary Figure 8. Characterization of *Eco*RV as PIFE-ruler. PIFE in 1bp distance between *Eco*RV and dsDNA labeled with Cy3 as donor fluorophore. The acceptor ATTO647N is positioned on the opposite 5'-end in 40 bp distance. **(A-B)** Background-corrected 2D histograms: Binding of *Eco*RV to free DNA results in a shift from S = 0.30 (unbound, black circle) to 0.39 (bound species, blue circle) for different concentrations. The conformation probed by the proximity ratio E_{Pr} stays unaltered. **(C)** Full data set of the *Eco*RV ruler presented in Figure 4D.

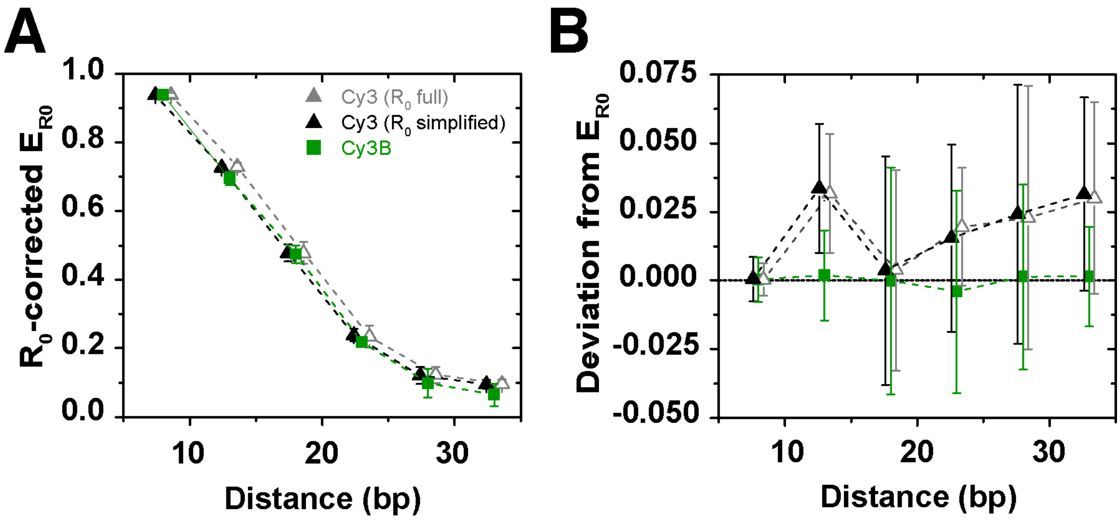

Supplementary Figure 9. Comparison of accurate FRET values of Cy3- and Cy3B-labelled DNA using different correction procedures. (A) Accurate FRET values for Cy3b (squared) and Cy3 (triangle). Using the proposed full (black) or simplified (grey) R_0-correction procedure for Cy3 labelled dsDNA, accurate FRET values of Cy3 can be converted to the R_0-axis of Cy3b. The employed dsDNA was labelled with the donor at the 5'-end and ATTO647N in 8,13, 18, 23, 28 and 33bp distance (see Fig. S4A). **(B)** Difference between accurate FRET values for Cy3 (triangle) compared to Cy3b after full (black) and simplified (grey) R_0-correction. The deviation between both methods is shown in green squares. Curves are shifted by 1bp to left and right for better visibility.

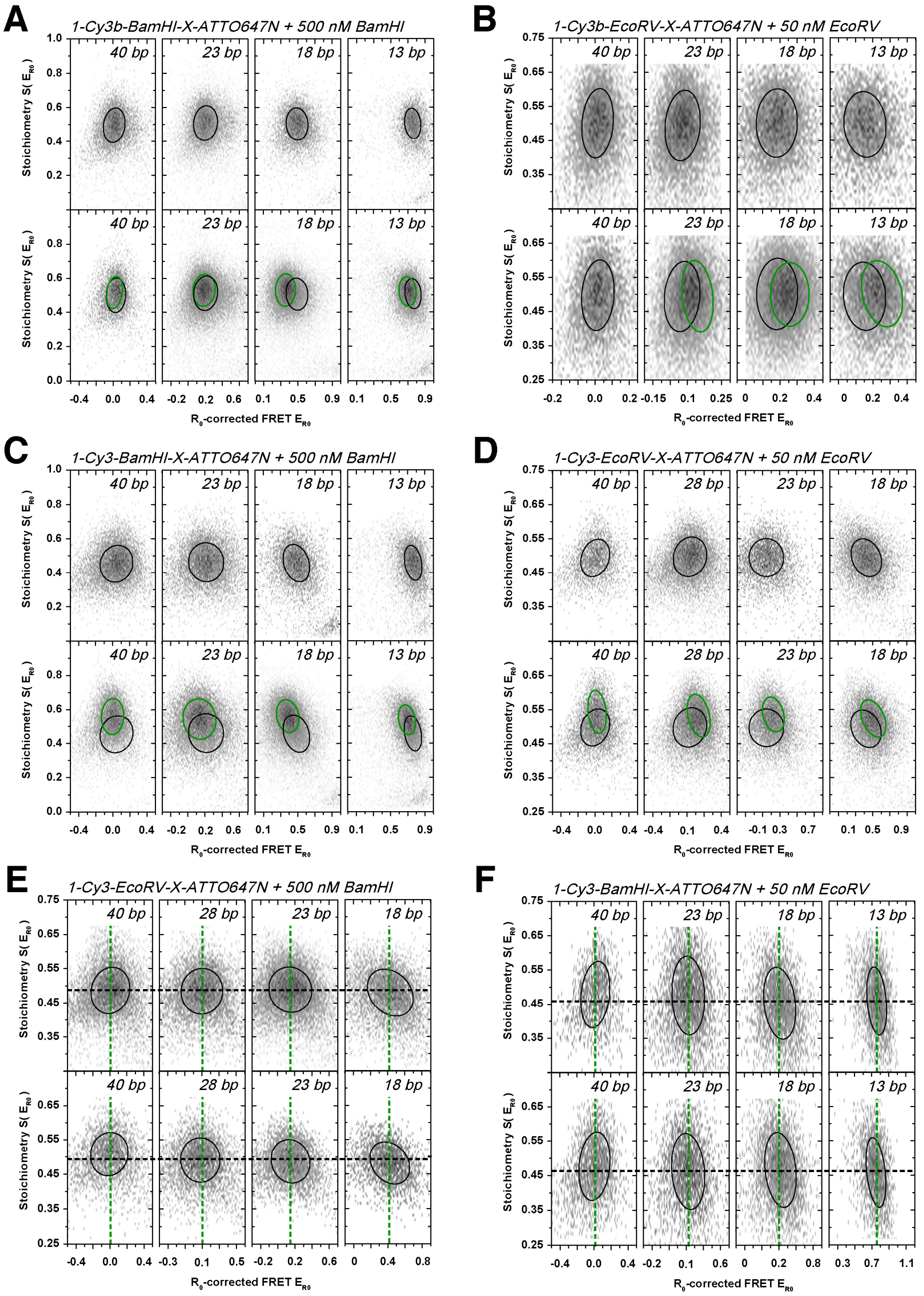

Supplementary Figure 10. Characterization of PIFE in the presence of FRET. 2D histograms of 40bp-dsDNA in absence and presence of *Bam*HI or *Eco*RV. The variable position of ATTO647N is mentioned in each 2D histogram. **(A-B)** Control with Cy3B as donor at the 5'-end. While *Bam*HI is not interfering with the protein structure, binding of *Eco*RV leads to a kink of the DNA seen by an increase in FRET. Binding and hence a shift in Stoichiometry is not observed. Full data set underlying Figure 6C and E. **(C-D)** Control with Cy3 as donor at the 5'-end. Compared to Cy3B (Fig. S10A-B), binding is additionally detected for both proteins by a discrete increase in Stoichiometry. Full data set presented in Figure 6C and E. **(E-F)** Specific binding of *Bam*HI and *Eco*RV. Both restriction enzymes bind exclusively to their palindromic sequence. *Bam*HI does not bind to 40bp-dsDNA carrying the sequence for *Eco*RV, nor *Eco*RV to dsDNA containing a recognition site for *Bam*HI.

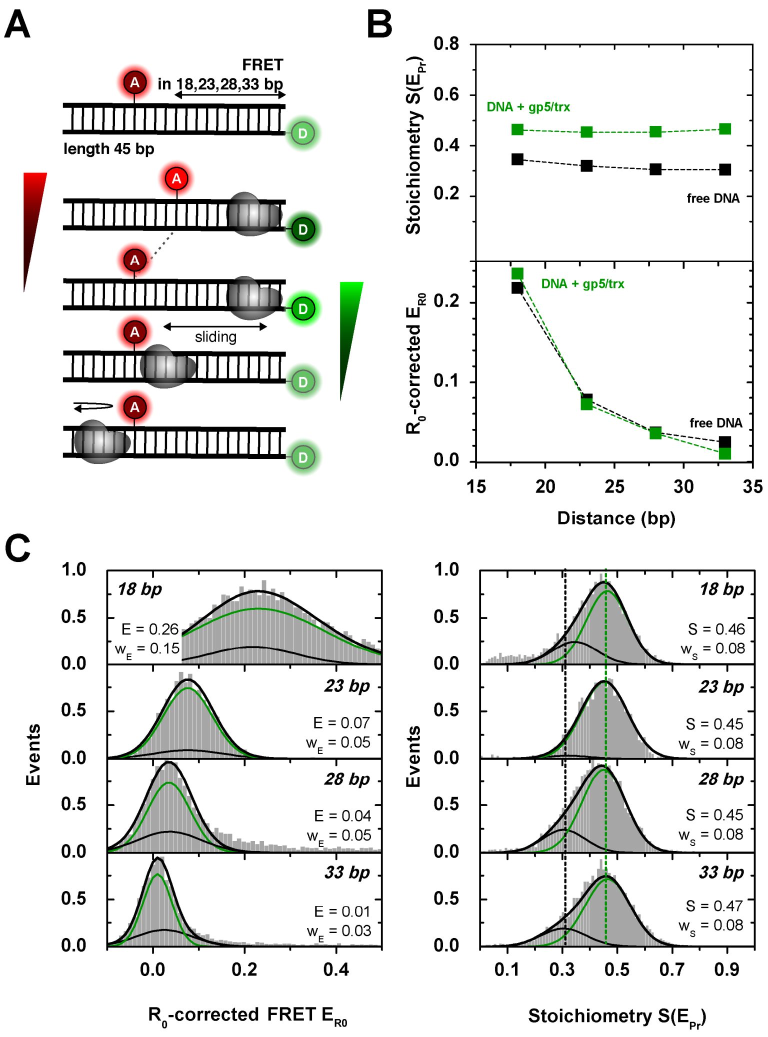

Supplementary Figure 11. FRET-distance dependent PIFE effect of T7 DNA polymerase gp5/trx on dsDNA. (A) Labelling scheme of the applied assay. 45bp long dsDNA oligos are labelled with Cy3 at the 5' and and ATTO647N in 18,23,28 and 33bp. Gp5/trx binds unspecific to dsDNA and slides along[4] from 5' to 3'-end. Depending on the position of ATTO647N two binding positions are possible: either between the two fluorophores or at the opposite 5'-end. Only centred binding allows for PIFE enhancement of Cy3. **(B)** Binding to 18,23,28 and 33bp-distal dsDNA labelled with Cy3/ATTO647N leads to an enhancement of Cy3 and hence a shift in Stoichiometry $S(E_{Pr})$. R_0-correction of the data gives evidence, that no structural alteration due to binding of gp5/trx has occurred and proves our data analysis strategy. **(C)** Full data set. 1D-histograms of R_0-corrected FRET E and Stoichiometry $S(E_{Pr})$ with projections of the applied 2D-fit functions. No conformational changes are detected. Binding of gp5/trx leads to an identical enhancement for all measured FRET distances R_2.

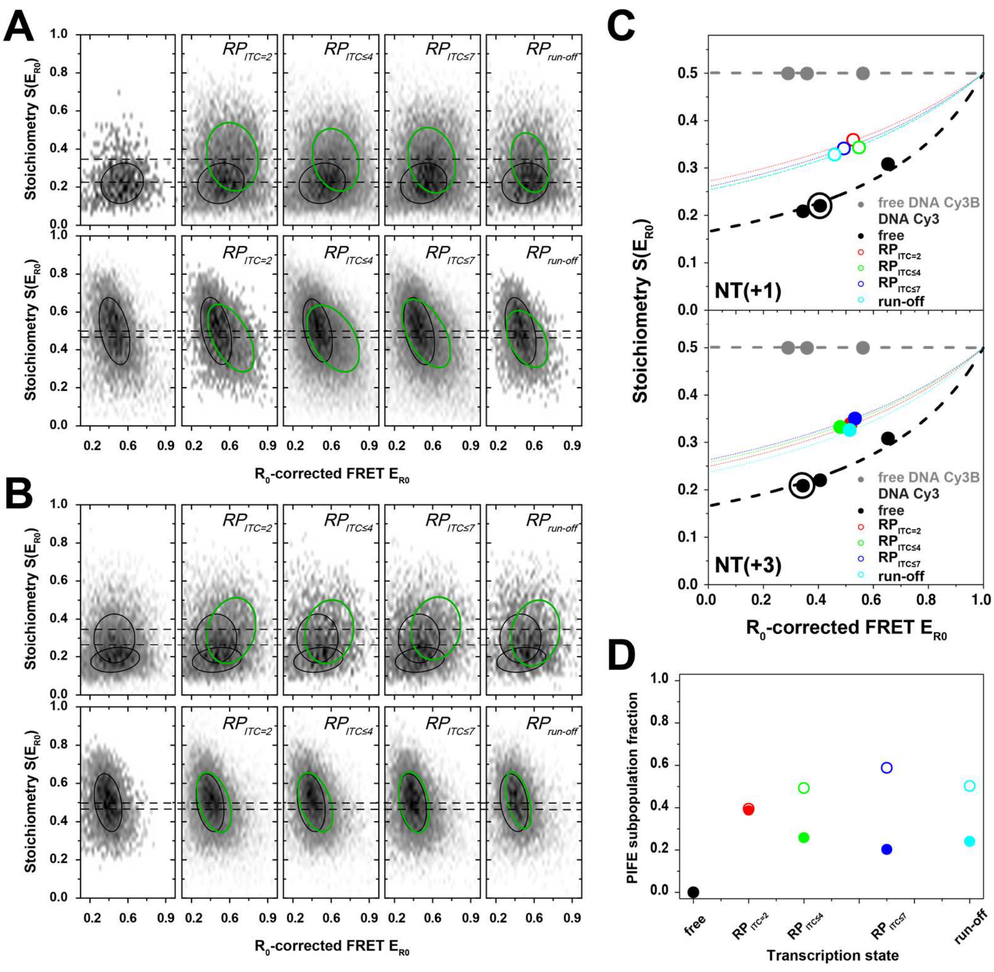

Supplementary Figure 12. Correction procedure to decouple FRET and PIFE occurring between double-labeled dsDNA and RNAP during transcription initiation. (A-B) Full data set of Figure 7 showing the 2D E-S histogram after R_0-correction for the registers -15/+1 **(A)** and -15/+3 **(B)** labeled with Cy3(B) and ATTO647N. The employed DNA sequences are provided in Supplementary Figure S4F. R_0-corrected E and S are retrieved by 2D Gaussian fitting. (**C**) Fitting procedure to retrieve the PIFE enhancement factor ρ as described in[5]. The determined R_0-corrected E and S values for free dsDNAs and DNAs bound to the RNAP in different transcription states are plotted against each other. Free dsDNAs labeled with Cy3B (grey) and Cy3 (black) are depicted for registers -15/+1, -15/+3 and +10/-6 for a better fit. dsDNA bound to the RNAP in different transcription states RP_{ITCsi} are depicted in color in the register +15/+1 (top) and +15/+3 (bottom). (**D)** Ratio of free dsDNA and dsDNA bound to RNAP for the registers +15/+1 (filled circles) and +15/+3 (open circles). After supplementing all NTPs, full transcription is expected to occur, after which, RNAP and the full transcript dissociate from the promoter and the transcription bubble is re-annealed. Therefore, a FRET population, more similar to that of the free promoter is expected. However, as observed in Fig. S12D, an RNAP-bound subpopulation with PIFE characteristics, still exists, even after supplementing RNAP with all four NTPs. This may imply that the labeled lacCONS promoter does not allow a full run-off and may get RNAP into being "stuck". Nevertheless, the number of RNAP molecules that ran-off the promoter can be inferred from the PIFE subpopulation fraction shown in Fig. S12D. It can be seen that the fraction of the PIFE subpopulation decreases in run-off, in comparison to the other states involved with the presence of RNAP and NTPs.

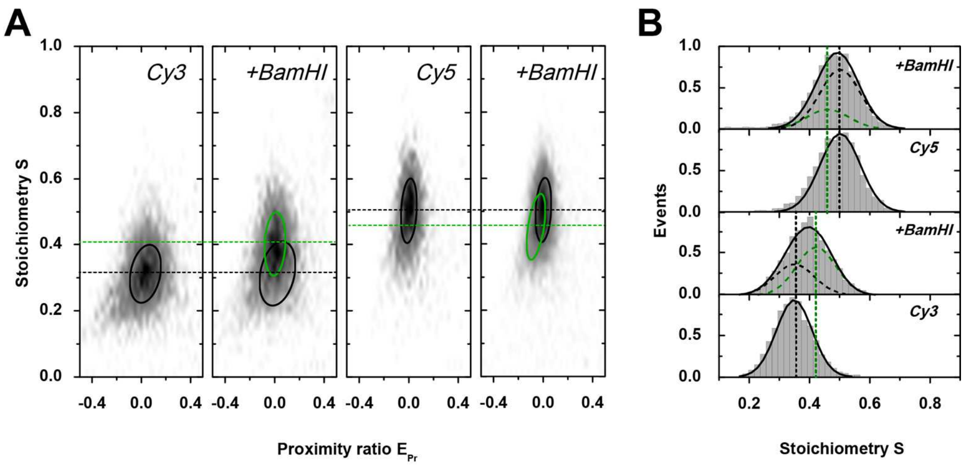

Supplementary Figure 13. Donor versus acceptor-based PIFE-ALEX. (A) Cy3-Atto647n and TMR-Cy5 labeled dsDNA with 40bp separation in the absence and presence of 500 nM *Bam*HI. 2D Gaussian fitting was applied to characterize the observed populations; black/green circles mark the FWHM of each distribution in the presence (green) and absence (black) of brightness changes of the donor Cy3 or acceptor Cy5. **(B)** 1D-histograms of Stoichiometry S(E_{Pr}) with projections of the applied 2D-fit functions. Binding of *Bam*HI leads to an increase in stoichiometry for Cy3 and a decrease for Cy5.

Supplementary Tabular 1. ATTO647N lifetime is mostly unaffected by binding to *Bam*HI. Fluorescence lifetime τ of ATTO647N attached to double-labeled dsDNA in the presence and absence of *Bam*HI. The donor Cy3(B) was attached to the 5'-end. The sample concentration was recorded at 5 nM for 2 min integration time. The excitation wavelength was set to 640 nm excitation at 1µW power. The fit-error amounts to 0.05 ns.

Cy3	Donor	PIFE / bp	FRET / bp	τ(ns) – free DNA	τ(ns) – bound DNA
dsB1	5'	1	40	4.28	4.31
dsB4	5'	2	40	4.21	4.32
dsB5	5'	3	40	4.22	4.26
dsB6	5'	5	40	4.20	4.23
dsB3	5'	7	40	4.30	4.41
dsB1	5'	1	40	4.28	4.31
dsB7	5'	1	23	4.03	4.13
dsB10	5'	1	18	3.97	4.01
Cy3B	Donor	PIFE / bp	FRET / bp	τ(ns) – free DNA	τ(ns) – bound DNA
dsB2	5'	1	40	4.20	4.25
dsB9	5'	1	23	4.04	4.13
dsB12	5'	1	18	3.91	4.04

Supplementary Table 2. Determined FRET and Stoichiometry values for dsDNA free and bound to *Bam*HI. Column 2 denotes the position of the donor, column 3, the position of the restriction enzyme and column 4 the position of the acceptor ATTO647N. Stoichiometry $S(E_{Pr})$ is provided after background and crosstalk-correction. R_0-corrected accurate FRET values E_{R0}, were referenced onto Cy3b with a gamma of 0.81. The applied donor fluorophores is mentioned in the corresponding header.

Cy3	Donor	PIFE / bp	FRET / bp	*Bam*HI	gamma	E_{Pr}	w_{0Pr}	$S(E_{Pr})$	$w_{S(EPr)}$
dsB1	5’	1	40	-	-	0.002	0.114	0.297	0.001
dsB4	5’	2	40	-	-	0.000	0.114	0.299	0.001
dsB5	5’	3	40	-	-	0.014	0.116	0.294	0.000
dsB6	5’	5	40	-	-	0.002	0.114	0.297	0.001
dsB3	5’	7	40	-	-	0.000	0.114	0.299	0.001
dsB1	5’	1	40	500 nM	-	0.003	0.078	0.386	0.005
dsB4	5’	2	40	500 nM	-	0.000	0.088	0.369	0.013
dsB5	5’	3	40	500 nM	-	0.000	0.078	0.354	0.007
dsB6	5’	5	40	500 nM	-	0.000	0.097	0.344	0.001
dsB3	5’	7	40	500 nM	-	0.037	0.091	0.280	0.013
**Cy3**	**Donor**	**PIFE / bp**	**FRET / bp**	***Bam*HI**	**gamma**	E_{R0}	w_{ER0}	$S(E_{Pr})$	$w_{S(EPr)}$
dsB1	5’	1	40	-	1.81	0.0439	0.164	0.313	0.082
dsB7	5’	1	23	-	2.08	0.2139	0.175	0.308	0.086
dsB10	5’	1	18	-	2.08	0.485	0.132	0.341	0.083
dsB13	5’	1	13	-	2.08	0.763	0.087	0.393	0.082
dsB1	5’	1	40	500 nM	1.92	0.003	0.111	0.405	0.081
dsB7	5’	1	23	500 nM	2.20	0.109	0.128	0.362	0.096
dsB10	5’	1	18	500 nM	2.20	0.389	0.117	0.417	0.076
dsB13	5’	1	13	500 nM	2.20	0.696	0.088	0.453	0.072
**Cy3**	**Donor**	**PIFE / bp**	**FRET / bp**	***Bam*HI**	**gamma**	E_{R0}	w_{ER0}	$S(E_{Pr})$	$w_{S(EPr)}$
dsB24	3	5	40	-	1.74	0.038	0.145	0.343	0.090
dsB25	3	5	23	-	2.00	0.208	0.132	0.331	0.079
dsB26	3	5	18	-	2.00	0.285	0.147	0.335	0.084
dsB27	3	5	13	-	2.00	0.581	0.138	0.379	0.083
dsB24	3	5	40	500 nM	1.88	0.024	0.091	0.402	0.089
dsB25	3	5	23	500 nM	2.16	0.162	0.103	0.374	0.081
dsB26	3	5	18	500 nM	2.16	0.233	0.119	0.382	0.077
dsB27	3	5	13	500 nM	2.16	0.526	0.110	0.425	0.072
**Cy3b**	**Donor**	**PIFE / bp**	**FRET / bp**		**gamma**	E_{R0}	w_{ER0}	$S(E_{Pr})$	$w_{S(EPr)}$
dsB2	5’	1	40	-	***0.81***	0.016	0.103	0.578	0.083
dsB9	5’	1	23	-	***0.81***	0.206	0.119	0.553	0.084
dsB12	5’	1	18	-	***0.81***	0.490	0.108	0.533	0.080
dsB14	5’	1	13	-	***0.81***	0.758	0.081	0.522	0.076
dsB2	5’	1	40	500 nM	***0.81***	0.021	0.079	0.580	0.073
dsB9	5’	1	23	500 nM	***0.81***	0.187	0.111	0.524	0.094
dsB12	5’	1	18	500 nM	***0.81***	0.357	0.099	0.544	0.085
dsB14	5’	1	13	500 nM	***0.81***	0.694	0.085	0.528	0.079

Supplementary Table 3. Determined FRET and Stoichiometry values for dsDNA free and bound to *Eco*RV. Column 2 denotes the position of the donor, column 3, the position of the restriction enzyme and column 4 the position of the acceptor ATTO647N. Stoichiometry $S(E_{Pr})$ is provided after background and crosstalk-correction. R_0-corrected accurate FRET values E_{R0}, were referenced onto Cy3B with a gamma of 0.81.

Cy3	Donor	PIFE / bp	FRET / bp	*Eco*RV	gamma	E_{Pr}	w_{EPr}	$S(E_{Pr})$	$w_{S(EPr)}$
dsE1	5’	1	40	-	-	0.019	0.108	0.300	0.077
dsE9	5’	2	40	-	-	0.037	0.116	0.318	0.081
dsE10	5’	3	40	-	-	0.053	0.103	0.292	0.061
dsE11	5’	5	40	-	-	0.032	0.099	0.318	0.070
dsE1	5’	1	40	50 nM	-	0.009	0.073	0.402	0.081
dsE9	5’	2	40	50 nM	-	0.000	0.071	0.402	0.079
dsE10	5’	3	40	50 nM	-	0.000	0.096	0.348	0.079
dsE11	5’	5	40	50 nM	-	0.022	0.090	0.334	0.063
Cy3	Donor	PIFE / bp	FRET / bp	*Eco*RV	gamma	E_{R0}	w_{ER0}	$S(E_{Pr})$	$w_{S(EPr)}$
dsE1	5’	1	40	-	***1.80***	0.016	0.155	0.306	0.087
dsE2	5’	1	28	-	***2.03***	0.082	0.169	0.288	0.091
dsE3	5’	1	23	-	***2.03***	0.190	0.172	0.296	0.088
dsE4	5’	1	18	-	***2.03***	0.430	0.177	0.329	0.088
dsE1	5’	1	40	50 nM	***1.02***	0.005	0.106	0.407	0.100
dsE2	5’	1	28	50 nM	***1.15***	0.132	0.108	0.370	0.089
dsE3	5’	1	23	50 nM	***1.15***	0.223	0.118	0.377	0.084
dsE4	5’	1	18	50 nM	***1.15***	0.513	0.120	0.382	0.078
Cy3b	Donor	PIFE / bp	FRET / bp	*Eco*RV	gamma	E_{R0}	w_{ER0}	$S(E_{Pr})$	$w_{S(EPr)}$
dsE5	5’	1	40	-	***0.72***	0.010	0.092	0.575	0.090
dsE6	5’	1	28	-	***0.81***	0.064	0.100	0.547	0.094
dsE7	5’	1	23	-	***0.81***	0.170	0.116	0.547	0.090
dsE8	5’	1	18	-	***0.81***	0.445	0.119	0.524	0.090
dsE5	5’	1	40	50 nM	***0.72***	0.014	0.078	0.584	0.080
dsE6	5’	1	28	50 nM	***0.81***	0.156	0.080	0.557	0.079
dsE7	5’	1	23	50 nM	***0.81***	0.265	0.093	0.532	0.087
dsE8	5’	1	18	50 nM	***0.81***	0.545	0.105	0.539	0.081

Supplementary Table 4. Distance uncertainty derived from steady-state anisotropy.

State	Dye / position	p donor	p min. donor	p max. donor	r'/r min.	r'/r max.
free	external Cy3	0.311	0.279	0.342	0.87	1.24
free	internal Cy3	0.241	0.195	0.286	0.88	1.21
free	external Cy3B	0.262	0.253	0.270	0.88	1.21
BamHI	external Cy3	0.344	0.305	0.382	0.87	1.24
BamHI	internal Cy3	0.279	0.224	0.332	0.88	1.21
BamHI	external Cy3B	0.315	0.276	0.353	0.87	1.24
free	internal atto 647N	0.156	0.137	0.175		
free	internal atto 647N	0.156	0.137	0.175		
free	internal atto 647N	0.156	0.137	0.175		
BamHI	internal atto 647N	0.208	0.183	0.232		
BamHI	internal atto 647N	0.208	0.183	0.232		
BamHI	internal atto 647N	0.208	0.183	0.232		

Supplementary Table 5. Determined FRET and Stoichiometry values for lacCONs DNA, free and bound to RNAP for the registers -15/+1 and -15/+3.

Cy3	Donor	Acceptor	gamma	E_{R0}	w_{ER0}	$S(E_{R0})$	$w_{S(ER0)}$
Free	NT: +1	T: -15	***0.84***	0.407	0.177	0.22	0.090
$RP_{ITC=2}$	NT: +1	T: -15	***0.84***	0.527	0.232	0.359	0.152
$RP_{ITC\leq4}$	NT: +1	T: -15	***0.84***	0.548	0.209	0.343	0.139
$RP_{ITC\leq7}$	NT: +1	T: -15	***0.84***	0.493	0.214	0.341	0.146
$RP_{Run\text{-}off}$	NT: +1	T: -15	***0.84***	0.459	0.170	0.328	0.132
**Cy3b**	**Donor**	**Acceptor**	**gamma**	E_{R0}	w_{ER0}	$S(E_{R0})$	$w_{S(ER0)}$
Free	NT: +1	T: -15	***0.84***	0.359	0.138	0.499	0.151
$RP_{ITC=2}$	NT: +1	T: -15	***0.84***	0.513	0.207	0.462	0.151
$RP_{ITC\leq4}$	NT: +1	T: -15	***0.84***	0.521	0.234	0.457	0.150
$RP_{ITC\leq7}$	NT: +1	T: -15	***0.84***	0.434	0.218	0.487	0.154
$RP_{Run\text{-}off}$	NT: +1	T: -15	***0.84***	0.427	0.185	0.458	0.128
**Cy3**	**Donor**	**Acceptor**	**gamma**	E_{R0}	w_{ER0}	$S(E_{R0})$	$w_{S(ER0)}$
Free	NT: +3	T: -15	***0.84***	0.345	0.208	0.209	0.104
$RP_{ITC=2}$	NT: +3	T: -15	***0.84***	0.518	0.218	0.339	0.147
$RP_{ITC\leq4}$	NT: +3	T: -15	***0.84***	0.480	0.219	0.332	0.143
$RP_{ITC\leq7}$	NT: +3	T: -15	***0.84***	0.533	0.222	0.350	0.141
$RP_{Run\text{-}off}$	NT: +3	T: -15	***0.84***	0.514	0.218	0.326	0.147
**Cy3b**	**Donor**	**Acceptor**	**gamma**	E_{R0}	w_{ER0}	$S(E_{R0})$	$w_{S(ER0)}$
Free	NT: +3	T: -15	***0.84***	0.291	0.125	0.499	0.128
$RP_{ITC=2}$	NT: +3	T: -15	***0.84***	0.332	0.155	0.501	0.132
$RP_{ITC\leq4}$	NT: +3	T: -15	***0.84***	0.333	0.156	0.501	0.130
$RP_{ITC\leq7}$	NT: +3	T: -15	***0.84***	0.307	0.137	0.501	0.135
$RP_{Run\text{-}off}$	NT: +3	T: -15	***0.84***	0.337	0.115	0.512	0.126

Supplementary Note 1 – **Obtaining accurate FRET values with full R_0-correction**

Quantum yield changes or spectral shifts of donor or acceptor fluorophore induced by PIFE or quenching have consequences for both the Förster radius R_0 and γ-factor. For a certain FRET pair, a change in the acceptor QY Φ_A has no consequences on R_0 but alters the γ-factor. The Förster radius R_0 is influenced by (i) the quantum yield changes Φ_D of the donor or (ii) changes of refractive index n. The ratio ℓ of Förster radii in presence R_0 and absence $R_{0;ref}$ of donor PIFE is given by

$$\ell = \left(\frac{R_0}{R_{0,ref}}\right)^6 = \frac{\Phi_D}{\Phi_{D,ref}} \cdot \frac{n_{ref}^4}{n^4} \cdot \frac{\kappa^2}{\kappa_{ref}^2} \cdot \frac{J(\lambda)}{J(\lambda)_{ref}} \qquad \textbf{(Eq. S1)}$$

In case of donor QY changes due to PIFE or quenching, not only the correct gamma-factors need to be used but the change in R_0 alters the accurate FRET value E, and hence has to be corrected to obtain true distances from ALEX experiments. In order to disentangle the contribution from changes in R_0 to the accurate FRET value E, we have to correct the accurate FRET value of each burst as follows

$$E = \frac{1}{1 + \left(\frac{r}{R_0}\right)^6} = \frac{1}{1 + \frac{1}{\ell} \cdot \left(\frac{r}{R_{0,ref}}\right)^6} \qquad \textbf{(Eq. S2)}$$

In case the physical distance r remains constant, we obtain the enhancement-corrected, accurate FRET value E_{R0} by expressing the ratio between distance and Förster radius $(r/R_{0,ref})^6$ by the altered accurate FRET value E for every individual burst via

$$E_{R0} = \frac{1}{1 + \left(\frac{r}{R_{0,ref}}\right)^6} = \frac{E}{E + \ell \cdot (1 - E)} \qquad \textbf{(Eq. S3)}$$

Depending on the applied fluorophores, fluorophore-linker, protein-interactions etc. different quantities can contribute to the ratio ℓ of Förster radii. As shown in

Supplementary Figure S2, the spectral characteristics of Cy3 and Cy3B are unaltered in the presence of a quencher or enhancer, only their amplitude varies. We measured the refractive index n of both solutions containing free or complexed DNA (data not shown). No difference in n until the 4th digit was observed. The presence of a protein binding close to Cy3, tethered by a C6-linker to the DNA, can influence κ^2 but anisotropy data shows that acceptor ATTO647N has free rotation and the assumption of a constant κ^2 remains valid. For the case of Cy3 and Cy3B, we suggest that ℓ can be simplified to

$$\ell \approx \frac{\Phi_D}{\Phi_{D,ref}} \qquad \textbf{(Eq. S4)}$$

The fold enhancement can be determined in ALEX experiment using the γ-values of PIFE and reference fluorophore. ATTO647N has been chosen as acceptor fluorophore in combination with Cy3 and Cy3B, where all show no spectral shift upon PIFE. The detection efficiencies η_i are assumed constant, so as the QY Φ_A of ATTO647N. Therefore, the ratio of γ's between the two fluorophore-pairs only depends on the donor's QY Φ_D

$$\frac{\gamma}{\gamma_{ref}} = \frac{\Phi_{D,ref}}{\Phi_D} \cdot \frac{\Phi_A}{\Phi_{A,ref}} \cdot \frac{\eta_D}{\eta_{D,ref}} \cdot \frac{\eta_{A,ref}}{\eta_A} \approx \frac{\Phi_{D,ref}}{\Phi_D} = \frac{1}{\ell} \qquad \textbf{(Eq. S5)}$$

Supplementary Note 2 – Obtaining accurate FRET values with simplified R_0-correction

The introduced s correction procedure treats Cy3-, Cy3-PIFE and Cy3B-based population with their corresponding γ-values. All data is referenced afterwards onto the Cy3B-axis via an R_0-correction, i.e., FRET efficiency E_{R0} translates into distances via the R_0 of Cy3B. Using Cy3 and Cy3B on dsDNA we emulated the PIFE effect and showed in Figure 5 that

$$E_{Cy3B} = E_{R0_{Cy3}} = \frac{E_{Cy3}}{E_{Cy3} + \ell(1 - E_{Cy3})} \qquad \textbf{(Eq. S6)}$$

with $$E_{Cy3} = \frac{F^{FRET}}{\gamma_{Cy3} \cdot f(DD) + F^{FRET}}$$

and $$\ell = \frac{\gamma_{Cy3B}}{\gamma_{Cy3}} \approx \frac{\Phi_{Cy3}}{\Phi_{Cy3B}}$$

This correction procedure, however, can get complicated, in case the gamma factors for different subpopulations are not directly accessible, e.g., when the original and PIFE population strongly overlap or one has low abundance. We show in the following that the procedure can be simplified to obtain R_0-corrected FRET value of any population of Cy3 where only the QY differs with respect to Cy3B. Instead of the correction outlined in eqns. 1-6, we suggest to determine accurate FRET values using γ_{Cy3B} in Step 2 without further corrections (see main text). The validity of this approach is shown by:

$$E_{Cy3b} = E_{R0_{Cy3}} \qquad \textbf{(Eq. S7)}$$

$$E_{R0_{Cy3}} = \frac{1}{1 + \ell\left(\frac{1}{E_{Cy3}} - 1\right)}$$

$$= \frac{1}{1 + \ell\left(\frac{\gamma_{Cy3} \cdot f(DD) + F^{FRET}}{F^{FRET}} - \frac{F^{FRET}}{F^{FRET}}\right)}$$

$$= \frac{1}{1 + \ell\left(\frac{\gamma_{Cy3} \cdot f(DD)}{F^{FRET}}\right)}$$

Which as has to be identical with

$$E_{Cy3B} = \frac{1}{1 + \ell_2 \left(\frac{\gamma_{Cy3B} \cdot f(DD)}{F^{FRET}} \right)}$$ **(Eq. S8)**

Resulting in $$\ell \cdot \gamma_{Cy3} = \ell_2 \cdot \gamma_{Cy3b}$$ **(Eq. S9)**

$$\ell_2 = \ell \cdot \frac{\gamma_{Cy3}}{\gamma_{Cy3B}} = \frac{\gamma_{Cy3B}}{\gamma_{Cy3}} \cdot \frac{\gamma_{Cy3}}{\gamma_{Cy3B}} = 1$$ **(Eq. S10)**

The consequence of eqns. S7-S10 is that any Cy3-labeled species that is corrected with gamma values of Cy3B is automatically normalized to the R_0-axis of Cy3B, in case Eqn. S4 is applicable.

References:

1. Eftink, M.R., Selvidge, L. A. . Fluorescence quenching of liver alcohol dehydrogenase by acrylamide. *Biochemistry* **21**, 117-125 (1982).
2. Mukhopadhyay, J. et al. Translocation of sigma(70) with RNA polymerase during transcription: fluorescence resonance energy transfer assay for movement relative to DNA. *Cell* **106**, 453-63 (2001).
3. Kim, S. et al. High-throughput single-molecule optofluidic analysis. *Nat Methods* **8**, 242-U83 (2011).
4. Etson, C.M., Hamdan, S.M., Richardson, C.C. & van Oijen, A.M. Thioredoxin suppresses microscopic hopping of T7 DNA polymerase on duplex DNA. *Proc Natl Acad Sci U S A* **107**, 1900-5 (2010).
5. Lerner, E., Ploetz, E., Hohlbein, J., Cordes, T. & Weiss, S. A Quantitative Theoretical Framework For Protein-Induced Fluorescence Enhancement-Forster-Type Resonance Energy Transfer (PIFE-FRET). *Journal of Physical Chemistry B* **120**, 6401-10 (2016).
